# Fuzzy Clustering Applied to ROI Detection in Helical Thoracic CT Scans with a New Proposal and Variants

**DOI:** 10.1155/2016/8058245

**Published:** 2016-07-18

**Authors:** Alfonso Castro, Alberto Rey, Carmen Boveda, Bernardino Arcay, Pedro Sanjurjo

**Affiliations:** ^1^Department of Information and Communication Technologies, Faculty of Computer Science, University of A Coruna, Campus de A Coruña, 15071 A Coruña, Spain; ^2^Radiology Service, Meixoeiro Hospital, Camiño Meixoeiro, 36200 Vigo, Spain

## Abstract

The detection of pulmonary nodules is one of the most studied problems in the field of medical image analysis due to the great difficulty in the early detection of such nodules and their social impact. The traditional approach involves the development of a multistage CAD system capable of informing the radiologist of the presence or absence of nodules. One stage in such systems is the detection of ROI (regions of interest) that may be nodules in order to reduce the space of the problem. This paper evaluates fuzzy clustering algorithms that employ different classification strategies to achieve this goal. After characterising these algorithms, the authors propose a new algorithm and different variations to improve the results obtained initially. Finally it is shown as the most recent developments in fuzzy clustering are able to detect regions that may be nodules in CT studies. The algorithms were evaluated using helical thoracic CT scans obtained from the database of the LIDC (Lung Image Database Consortium).

## 1. Introduction

In the field of medical image analysis, the thorax area has been the object of extensive investigation [1] due to the complexity of the pulmonary structure itself, with approximately 23 generations of branching arteries, and the problems experienced in the detection of elements of interest within this structure (nodules, tumours, etc.) [2].

The most widely used images for diagnosis have traditionally been chest X-rays because of their low cost. However, images obtained using helical CTs are being used more and more since they enable high-definition observation of lung structures, allowing images to be acquired in intervals of time shorter than a breath and with resolutions of less 1 mm. It is becoming increasingly possible to find multislice CTs [3] which provide a more accurate image of the area under examination, although they are rather costly and still not very widespread.

**Figure 1:**
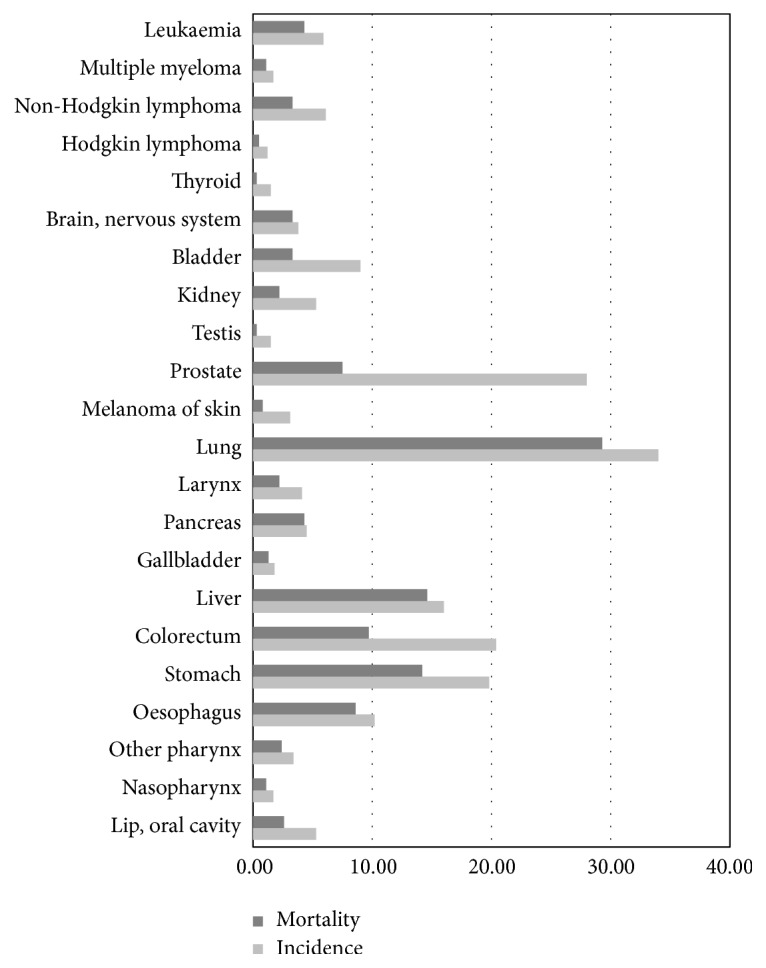
Incidence and mortality of different types of cancer in men.

Within this field, one of the problems that has received most attention is the detection of pulmonary nodules due to the high rates of lung cancer found in modern societies. This disease has one of the highest mortality rates (Figure 1, [4]) and therefore early detection is fundamental [5].

The analysis of these types of studies is extremely time consuming for the radiologist because of the huge amount of data that has to be analyzed (more than 100 thin-section images) [6] and also due to the difficulty in distinguishing nodules in their initial phase because they are not clearly defined and due to their similarity to other elements present in the lungs. Clinically speaking, a solitary pulmonary nodule is considered to be any isolated and intrapulmonary lesion, rounded or oval in shape, surrounded by ventilated lung, whose diameter according to arbitrarily established criteria is less than 4 cm [7]. Furthermore, the contours of a nodule or mass must also be sufficiently defined and clear in order to be able to determine its approximate size with relative precision.

On the basis of the aforementioned information, multiple CAD (Computer Aided Diagnosis) systems have been developed to perform this task with a wide variety of techniques being used for this purpose: [8] proposed a multilevel thresholding technique designed to identify connected components of similar intensity and eliminate vessels present in the CT in order to detect nodules; [9] divided the CT into grids using a genetic algorithm that used a template to detect elements which could correspond with nodules; [10] proposed a new QCI filter as part of a CAD to detect nodules in CT; and [11] used thresholding and morphological operators to detect candidate nodules followed by the use of a Fisher Linear Discriminant classifier to reduce false positives. Other papers describing major systems within this area are [12–19].

Our research group is developing a CAD system to perform this task automatically. This system uses fuzzy logic as a basis for detecting lung nodule candidates and, in particular, fuzzy clustering algorithms.

**Figure 2:**
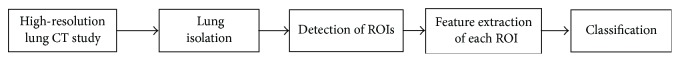
Phases of a CAD system.

**Figure 3:**
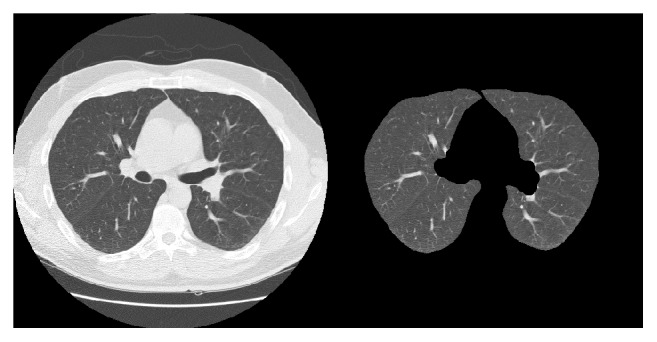
Original CT image and the result after applying the preprocessing stage to isolate the lungs.

In Figure 2 we can see the phases of a typical CAD system. The first task to be undertaken in pulmonary CAD systems is a preprocessing stage to isolate the pulmonary lobes, removing external elements that may affect classification. The system we are developing also includes an initial stage for this purpose [20, 21], Figure 3. In this process each of the unwanted elements (e.g., the diaphragm) is isolated and eliminated in a series of steps and when the only remaining elements are the lungs themselves, a range of morphological operations (opening, closing) are applied to eliminate any defects that might have arisen during the process, such as the recuperation of pixels previously eliminated from the juxtapleural nodules.

This work focuses on the following phase, the purpose of which is to detect ROIs with a view to reducing the search area and obtaining the lowest possible number of candidate zones that may be nodules; the aim is to reduce the number of false positives and increase that of true positives. The objective is for this stage to be conducted automatically by the system given its advantages: a significant reduction in the workload of the specialist and the elimination of bias errors.

In this paper, we present and analyze the results of various fuzzy clustering algorithms that use different strategies to classify the pixels that make up an image. We also propose a new algorithm, formulated by merging two of the algorithms we have analyzed.

The FCM, KFCM, SFCM, and SKFCM algorithms were studied and the MSKFCM algorithm is proposed. The algorithms analyzed using spatial information were modified so that 3D neighborhoods could be used in the classification process (these algorithms were originally designed for use with 2D neighborhoods) which should allow for a better classification, working with further information, and offer a better reflection of the authentic anatomical structure.

Section 2 on material discusses the characteristics of the studies used in the tests and the tools employed to implement the algorithms. A description is then provided of each algorithm.  Section 3 describes the methodology used in the tests. In Section 4 we present and discuss the results obtained and the metrics used to take measurements. Finally, the conclusions will be considered.

## 2. Material and Methods

For the purposes of this analysis we used a set of helical thoracic CT scans from the LIDC (Lung Image Database Consortium) [22], which can be accessed from the National Biomedical Imaging Archive (NBIA).

The goal of this project is to develop a reference repository of CT lung images for the development and evaluation of CAD systems in the detection of lung nodules. Five North American institutions have collaborated in its construction: Cornell University; the University of California, Los Angeles; the University of Chicago; the University of Iowa; and the University of Michigan.

Each image was annotated by four experts, initially as a blind review, so that any discrepancies between annotations could then be forwarded to the corresponding experts, who could then make the appropriate amendments. The images are stored according to the DICOM standard, sized 512 × 512, with a pixel size from 0.5 to 0.8 mm and a 12-bit grayscale of 12 bits in Hounsfield Units (HU). These CT scans were acquired from a wide range of scanner manufacturers and models under X-ray tube current ranging from 40 to 627 mA (mean: 221.1 mA) and tube voltage at either 120 or 140 kVp. The CT studies were reconstructed with pixel resolution ranging from 0.461 to 0.977 mm (mean: 0.688 mm) and slice thickness ranging from 0.45 to 5.0 mm (mean: 1.74 mm) [23].

Each analysis incorporates an XML file indicating the presence of one or more nodules (or their absence), their type, and their contour (specified by the coordinates of the constituent pixels).

**Figure 4:**
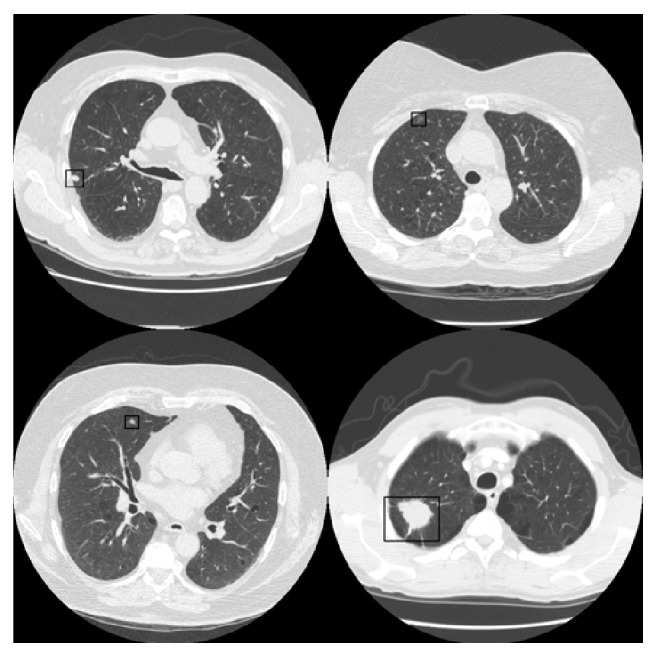
Different helical thoracic CT scans used in the tests with the nodule marked by a black rectangle.

Figure 4 shows some of the slices used in the study with the location of the nodule marked by a black rectangle.

Fuzzy clustering algorithms were used to detect ROIs due to their capacity for handling multidimensional information, making them easily adaptable for the classification of images, their low sensitivity to noise, which should make it easier to differentiate between nodules and other elements in images, and their capacity for handling ambiguous information, a common characteristic of medical images due to the low signal/noise ratio [24].

In the last years, new algorithms have been developed in order to resolve the problems associated with classical fuzzy algorithms and to provide better results [25–27]. In this paper, we selected some of the recent fuzzy algorithms that use kernel functions (KFCM) to simulate calculation in larger spaces or algorithms that use the pixel neighborhoods to calculate their membership (SFCM), increasing their insensitivity to noise. Moreover, those algorithms have been developed and tested within the medical image analysis field, being suitable to the problem described in this paper.

In this study, the two algorithms mentioned above were combined to obtain a new spatial kernelized algorithm and to determine whether the combination of these two techniques yielded better results than each technique individually for the problem addressed in this research. The SKFCM algorithm was analyzed to estimate the improvement that the new algorithm was expected to offer compared with the other algorithms using the same strategy. This has also been used for medical imaging analysis and specifically for MRI (Magnetic Resonance Imaging) in brain scans [28].

To enhance the quality and increase the scope of the analysis, the spatial algorithms were modified so that 3D neighborhoods could be used. These neighborhoods enabled a better appreciation of the real structure of the element to be defined and used more information in the classification process. For this reason, we expected to obtain a better classification than by using 2D neighborhoods.

We also analyzed the FCM algorithm which was the first fuzzy clustering algorithm to be developed and is currently used as a reference in the literature.

The ITK toolkit was used to implement the algorithms. This is an open-source software toolkit for registering and segmenting medical images, developed in C++ using the generic programming paradigm. The algorithms were implemented using base classes since there was no support for fuzzy logic.

Details of the implementation of some algorithms used in this analysis were published in Insight Journal [29] and are freely available to any interested researchers to allow the scientific community to confirm that the algorithms were implemented correctly and facilitate their use.

### 2.1. FCM (Fuzzy C-Means)

The FCM algorithm was developed by Bezdek et al. [30] and is the first fuzzy clustering algorithm. It is a method for the division of sets based on Picard iterations on the necessary conditions for calculating the minimum square error of the objective function:

(1) $$\begin{array}{c} J_{m}=\overset{n}{\underset{k=1}{\sum }}\overset{c}{\underset{i=1}{\sum }}{\left(\;u_{ik}\;\right)}^{m}{\left|\;x_{k}-v_{i}\;\right|}^{2}. \end{array}$$

In this algorithm, *u*_*ik*_ represents the membership value of pixel *k* to class *i*, *x*_*k*_ is the *k*th pixel, *v*_*i*_ the centroid for class *i*, *c* is the number of clusters, *n* is the number of pixels to classify, and *m* is a weight factor that must be bigger than 1. The FCM initially needs the number of clusters in which the image will be divided and a sample of each cluster.

The steps of this algorithm are as follows.

(1) Calculation of the membership of each element to each cluster:
  (2) uki,j=∑j=1cyi,j−vkyi,j−vj2/m−1−1,1≤k≤c,  1≤i,j≤n,
 where *y*(*i*, *j*) represents each pixel of the initial image.
(2) Calculation of the new centroids of the image:
  (3) $$\begin{array}{c} v_{k}=\frac{\sum_{i,j}u_{k}{\left(i,j\right)}^{m}y\left(i,j\right)}{\sum_{i,j}u_{k}{\left(i,j\right)}^{m}},\;k=1,\ldots ,c. \end{array}$$
(3) If the error stays below a certain threshold, stop. In the contrary case, return to step (1). The parameters that were varied in the analysis of the algorithm were the samples provided and the value of *m*.

### 2.2. KFCM (Kernelized Fuzzy C-Means)

This algorithm was proposed in Chen and Zhang [31] and is based on FCM, integrated with a kernel function that allows the transfer of the data to a space with more dimensionality, which makes it easier to separate the clusters.

The purpose of the kernel function is to “simulate” the distances that would be obtained by transferring the points to a space with more dimensionality, which in most cases would imply exaggerated computational costs. The proposed objective function is

(4) $$\begin{array}{c} J_{m}=\overset{c}{\underset{i=1}{\sum }}\overset{n}{\underset{k=1}{\sum }}u_{ik}^{m}\left(\;1-K\left(\;x_{k},v_{i}\;\right)\;\right). \end{array}$$

The kernel functions used most often are polynomial functions (5) and Gaussian radial basis functions (6). Consider

(5) KX,Y=φX·φY=X·Y+bd,

(6) KX,Y=φX·φY=exp⁡−X−Y22σ2,

where *σ* is the sigma of the Gaussian function.

The algorithm consists of the following steps.

(1) Calculation of the membership function:
  (7) $$\begin{array}{c} u_{ik}=\frac{{\left(1-K\left(x_{k},v_{i}\right)\right)}^{-1/\left(m-1\right)}}{\sum_{j=1}^{c}{\left(1-K\left(x_{k},v_{j}\right)\right)}^{-1/\left(m-1\right)}}. \end{array}$$
(2) Calculation of the new kernel matrix *K*(*x*_*j*_, *v*_*k*_) and *K*(*v*_*k*_, *v*_*k*_):
  (8) $$\begin{array}{c} v_{i}=\frac{\sum_{k=1}^{n}u_{ik}^{m}K\left(x_{k},v_{i}\right)x_{k}}{\sum_{k=1}^{n}u_{ik}^{m}K\left(x_{k},v_{i}\right)}. \end{array}$$
(3) If the error stays below a determined threshold, stop. In the contrary case, return to step (1).

The different parameters for the analysis of this algorithm were the initial samples and number of clusters.

### 2.3. SFCM (Spatial Fuzzy C-Means)

This is a spatial fuzzy clustering algorithm [32] that uses a spatial function, which is the sum of the memberships of the pixels in the neighborhood of the pixel under consideration. The main advantages deriving from the use of a spatial function are the possibility of obtaining more homogeneous regions and less sensitivity to noise.

In the initial stage the algorithm applies the traditional FCM (Fuzzy C-Means) algorithm to obtain the initial memberships for each pixel, the iterative stage being omitted. It then calculates the spatial function value for each pixel in the image:

(9) $$\begin{array}{c} h_{ij}=\underset{k\in NB\left(x_{j}\right)}{\sum }u_{ik}, \end{array}$$

where NB(*x*_*j*_) represents a square window centred around the pixel under consideration, its size being a configurable parameter of the algorithm. The greater the number of neighboring pixels that belong to the same cluster, the higher the value of the function.

The next step is to calculate the spatial membership function:

(10) $$\begin{array}{c} u_{ij}^{\prime }=\frac{u_{ij}^{p}h_{ij}^{q}}{\sum_{k=1}^{c}u_{kj}^{p}h_{kj}^{q}}, \end{array}$$

where *p* and *q* are control parameters for the importance of functions *u*_*ij*_ and *h*_*ij*_. Finally, the new centroids are calculated:

(11) $$\begin{array}{c} c_{j}=\frac{\sum_{i=1}^{N}u_{ij}^{\prime m}x_{i}}{\sum_{i=1}^{N}u_{ij}^{\prime m}}. \end{array}$$

The error is calculated. When this is below a determined threshold, the algorithm will stop; otherwise the FCM will be recalculated and a further iteration will commence.

### 2.4. SKFCM (Spatial Kernelized Fuzzy C-Means)

This algorithm [28] introduces a penalty factor that contains spatial neighborhood information to the KFCM (Kernelized Fuzzy C-Means) algorithm proposed in the same study. The paper only considers the Gaussian radial basis function kernel:

(12) $$\begin{array}{c} K\left(\;x,y\;\right)=\exp\left(\;-\frac{{\left\|x-y\right\|}^{2}}{\sigma^{2}}\;\right). \end{array}$$

Therefore, modifying the objective function of the FCM in order to introduce the kernel function and add the penalty factor, we obtain the final objective function:

(13) JSmϕ=∑i=1c∑k=1Nuikm1−Kxk,vi+αNR∑i=1c∑k=1Nuikm∑r∈Nk1−Kxr,vi,

where *N*_*k*_ represents the square window which includes the neighbors of pixel *x*_*k*_ (without considering it), *N*_*R*_ is the cardinality of *N*_*k*_, and *α*  (0 < *α* < 1) is a parameter that controls the effect of the penalty term. Deriving the objective function (see (13)) with respect to *u*_*ik*_ and *v*_*i*_, the authors obtained two conditions that minimize the objective function. Finally, an iterative algorithm can be derived from the above conditions.

When initialising the algorithm, the parameters *c*, that is, the number of clusters, the initial class centroids, and the threshold epsilon, must be determined.

In the first step of the iterative process, the memberships are calculated as follows:

(14) $$\begin{array}{c} u_{ik}=\frac{{\left(\left(1-K\left(x_{k},v_{i}\right)\right)+\left(\alpha /N_{R}\right)\sum_{r\in N_{k}}{\left(1-K\left(x_{r},v_{i}\right)\right)}^{m}\right)}^{-1/\left(m-1\right)}}{\sum_{j=1}^{c}{\left(\left(1-K\left(x_{k},v_{j}\right)\right)+\left(\alpha /N_{R}\right)\sum_{r\in N_{k}}\left(1-K{\left(x_{r},v_{j}\right)}^{m}\right)\right)}^{-1/\left(m-1\right)}}. \end{array}$$

Finally, the centroids are updated as follows:

(15) vi=∑k=1nuikmKxk,vixk+α/NR∑r∈NkKxr,vixr∑k=1nuikmKxk,vi+α/NR∑r∈NkKxr,vi.

As in the other algorithms, repeat these steps until condition ‖*v*_*i*−1_ − *v*_*i*_‖ ≤ *ϵ* is satisfied, where epsilon is a determined threshold.

### 2.5. MSKFCM (Modified Spatial Kernelized Fuzzy C-Means)

The modification proposed in this study ([29]) is a combination of the algorithms described previously (KFCM and SFCM) in order to combine their strengths. Thus, kernelized algorithms simulate the calculation of distances in a space of greater dimensionality, enabling better classification of elements. Spatial algorithms reduce sensitivity to noise and local variations by using the membership of all the pixels belonging to the neighborhood we wish to calculate.

The initial parameters required for the proposed modification are the number of clusters into which the image is to be divided, a sample of each cluster, and the values for the parameters *p*, *q* in order to calculate spatial membership.

The algorithm consists of the following steps.

(1) Calculation of the membership function:
  (16) $$\begin{array}{c} u_{ik}=\frac{{\left(1-K\left(x_{k},v_{i}\right)\right)}^{-1/\left(m-1\right)}}{\sum_{j=1}^{c}{\left(1-K\left(x_{k},v_{j}\right)\right)}^{-1/\left(m-1\right)}}. \end{array}$$
(2) Calculation of spatial memberships:
  (17) $$\begin{array}{c} u_{ik}^{\prime }=\frac{u_{ik}^{p}h_{ik}^{q}}{\sum_{j=1}^{c}u_{jk}^{p}h_{jk}^{q}} \end{array}$$
 with *h*_*ik*_ = ∑_*z*∈NB(*x*_*k*_)_ *u*_*iz*_, where NB is the neighborhood centred in *x*_*k*_.
(3) Calculation of the new centroids:
  (18) $$\begin{array}{c} v_{i}^{\prime }=\frac{\sum_{k=1}^{n}u_{ik}^{\prime m}K\left(x_{k},v_{i}\right)x_{k}}{\sum_{k=1}^{n}u_{ik}^{\prime m}K\left(x_{k},v_{i}\right)}. \end{array}$$
(4) If the error stays below a determined threshold ‖*v*_*i*−1_ − *v*_*i*_‖  ≤*ϵ*, stop. In the contrary case, return to step (1).

By combining what are currently the two most widely used techniques for developing fuzzy clustering algorithms, we aimed to improve the classification of the pixels forming the nodule, improve the detection of true positives using a kernel function to improve cluster separation, and reduce false positives using neighboring pixels to calculate membership. Consequently, it was determined that, with the exception of very fuzzy nodules, the pixels forming it had neighborhoods that allowed better differentiation from other areas of the image with similar values for each pixel.

## 3. Methodology

To carry out the analysis, a moderate number of studies were used to better determine the different features that influence the outcome and detect any possible problems that might arise. 1500 slices were used belonging to the nine studies which contain the different cases in this type of medical image: the initial stage, adherent to the pulmonary membrane, clearly consolidated, and located in the different thoracic zones: lower, middle, and upper.

In order to measure the success rate of the algorithms we decided to calculate the number of true positives (TP) and false positives (FP), sensitivity against sensibility, considering the true positive as those pixels that are part of the nodule and they are classified as nodule. Oppositely, false positives are pixels classified as nodule but really they are part of another element of the slice. An algorithm that correctly classifies the nodules must assign a high number of true positives and a low number of false positives. If other values were obtained, this would indicate that the algorithm performed a poor classification, either because the rate of success in terms of the classification of the nodule pixels was low or because the algorithm incorrectly classified a large number of pixels that were not nodule as nodule pixels.

The traditional method used to evaluate CAD systems is to use the outcome for a series of cases for which the results are known and to construct a ROC curve on the basis of TPF (True-Positive Fraction) and FPR (False-Positive Fraction) [33] so that the quality of the system can be observed as well as the outcome through the variation of different parameters. However, given the declared aim of this work, this is not the most adequate focus, given that the objective of this module is not to identify the ultimate outcome but to reduce the search space to localize those zones that may be nodules.

For this reason, we decided to use the approximation proposed by Bowyer [34] to evaluate edge detection algorithms. In this framework, each set of parameter values for each edge detector and image will produce a count of true-edge pixels and false-edge pixels. By sampling broadly enough in the parameter space for an edge detector, and at fine enough intervals, it is possible to produce a representative range of possible tradeoffs in true versus false positives. This results in a graphical representation of possible “true positive/false positives” tradeoffs similar to a receiver operating characteristic (ROC) curve. This provides a comparison of the behaviour of the algorithm for different parameters and the selection of the best combination, adapted and aligned to our aim.

Another factor that favored this solution for evaluating these results from fuzzy clustering algorithms was that the masks supplied by the LIDC for the different slices only contain information about the nodules indicating the points that constitute their edge and type, with no data on the other elements that may exist in each slice. The use of other measurements would involve creating masks with the correct classification for each pixel in each slice and for each study which is beyond the capacity of our group. Even so, we had to create an application using XML files that provides LIDC for each study with the data of the nodules for each slice and translates this information as a representation allowing for a rapid and efficient evaluation.

**Figure 5:**
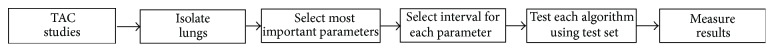
Flow chart followed to make the tests.

In Figure 5 the steps followed to make the tests can be seen. In first place, a preprocessing was applied to all the studies with the objective of isolating the lungs. In the next step, the relevant parameters were identified that influenced the results obtained for each algorithm. In the fourth step, we determine the test interval for each parameter of each algorithm, in order to reduce the search space. In this sense, different values were tested based on a fixed space covering the entire interval. Following that, we test each algorithm and the different combination of parameters over the data set. Finally, we evaluate the results obtained for each slice applying each algorithm with its combinations of parameters.

Table 1 shows the parameters analyzed for each algorithm and the ranges used for each parameter analyzed. The first parameter analyzed was the number of clusters into which the image was to be divided; the best results were obtained with three and four clusters; a different set of test images and different validity indices were used [35, 36]. The second parameters were the number and initial samples used for initialisation since these parameters could induce variations in algorithm convergence speed and results [37]. We decided to use samples that were obtained randomly and through an operator for each slice. Finally, it was observed that for parameters *p* and *q* of the SFCM and MKSFCM algorithms the best results were obtained in the interval [0,2].

To illustrate the results we will use graphs that allow us to see the conditions in which the best results were obtained for each algorithm. The aim is to estimate how stable they are and to visualize their behaviour for our studies. In order to improve the clarity in the presentation of the results, each algorithm will be presented using a different subsection.

## 4. Results

The first algorithm analyzed was the FCM due to its current status as a reference algorithm, as mentioned above.

### 4.1. FCM

**Figure 6:**
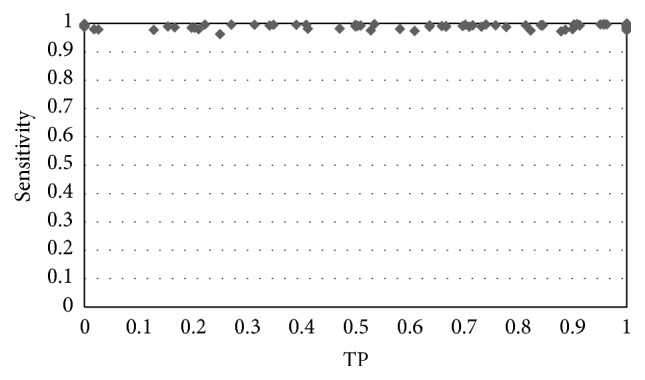
Results obtained for the FCM algorithm for one of the analyzed studies.

Figure 6 shows the results obtained for this algorithm in one study; the algorithm is used with samples selected by an operator (a radiologist) with each result represented by a point. For this algorithm, it was decided to represent the TP against sensitivity to better observe its behaviour. It can be observed in the graph that variability is quite high for the different slices: there are cases were the success rate is very low (below 40%) or very high (close to 100%); in addition the number of false positives is also high increasing with the success rate. The behaviour of the algorithm in the rest of the studies was similar.

This result is due to the FCM algorithm classifying by means of hyperspheres (if the Euclidean distance is used in the calculation of the memberships); it is not possible to separate mixed classes that have different structures [38], as is the present case, which impedes the algorithm from calculating centroids of sufficient quality to produce a good partitioning of the image. Further evidence that corroborates this fact is the results obtained using random samples, in which the values for TP and FP measurements were similar to those obtained using samples selected by an operator (the difference was less than 1%), which shows that the result principally depends on membership function used in the classification rather than the samples used.

**Figure 7:**
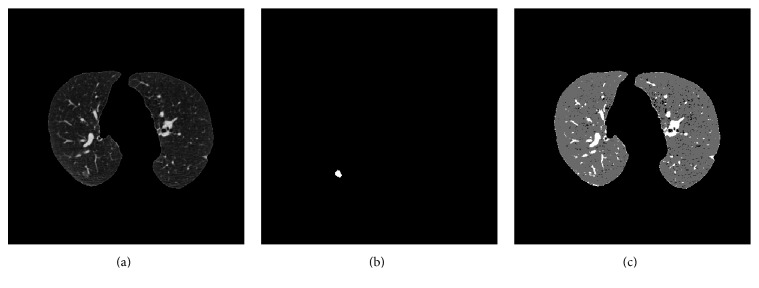
(a) Original image, (b) mask, and (c) result obtained for the FCM algorithm.

Figure 7 shows the result for the FCM algorithm for one of the slices used in the tests in which it can be observed that while the algorithm is able to detect a part of the nodule, there are an elevated number of false positives. The same slice will be used in the remainder of the paper to illustrate the results of the different algorithms and to facilitate their comparison.

### 4.2. KFCM

**Figure 8:**
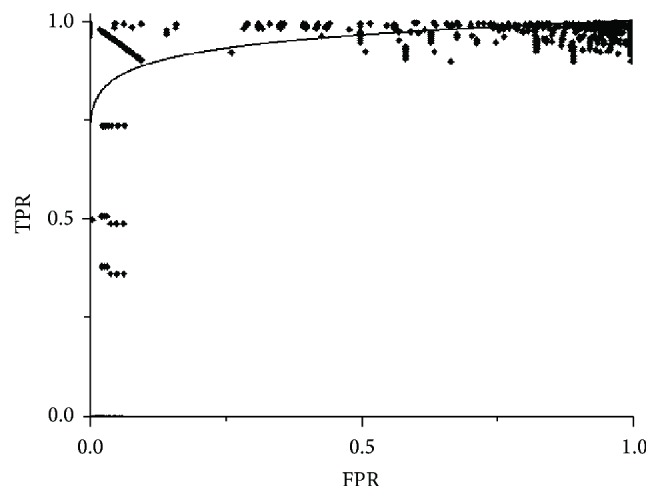
Results obtained for the KFCM algorithm for the different studies.

A Gaussian kernel was used in the testing process for the KFCM algorithm.  Figure 8 shows the results for this algorithm in all the analysis studies. In the graph, it can be observed that a high success rate for a significant number of slices was achieved. Nevertheless, the results indicate that this algorithm is not adequate for the automatic detection of ROIs, the aim of this paper. Although the success rate for the majority of slices is high (more than 65%), the noise level is very high (more than 30% in almost all). This can be clearly seen in the graph with the majority of points situated in the upper right corner making them very difficult to eliminate.

**Figure 9:**
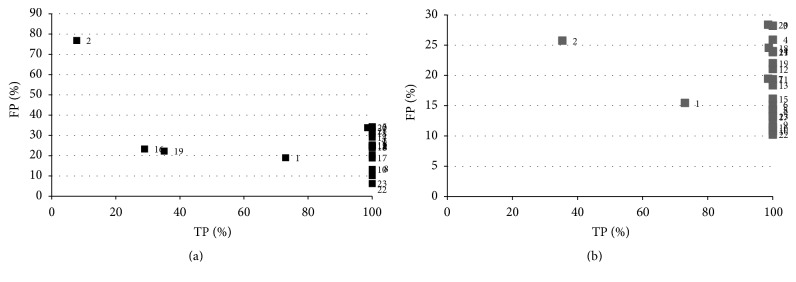
Results for a set of slices selected for the KFCM algorithm: (a) random samples, (b) operator samples.

Figure 9 shows the results obtained for 23 slices selected from all the studies analyzed in order to obtain a clearer insight into these results. This combination of slices was also used to illustrate the behaviour of the rest of the algorithms to allow for the comparison of the results and the performance of each algorithm. The graph shows how false positives reach 70% in various slices and are not lower than 20–30% in almost all. This implies that, even with the construction of an efficient classifier for the following stage, it would be extremely difficult to eradicate these erroneous zones from the result. An elevated number shows features that are very similar to those of a nodule, such as midrange HU values, shape, and size, which makes it very difficult to establish criteria that allow for a good classification.

**Figure 10:**
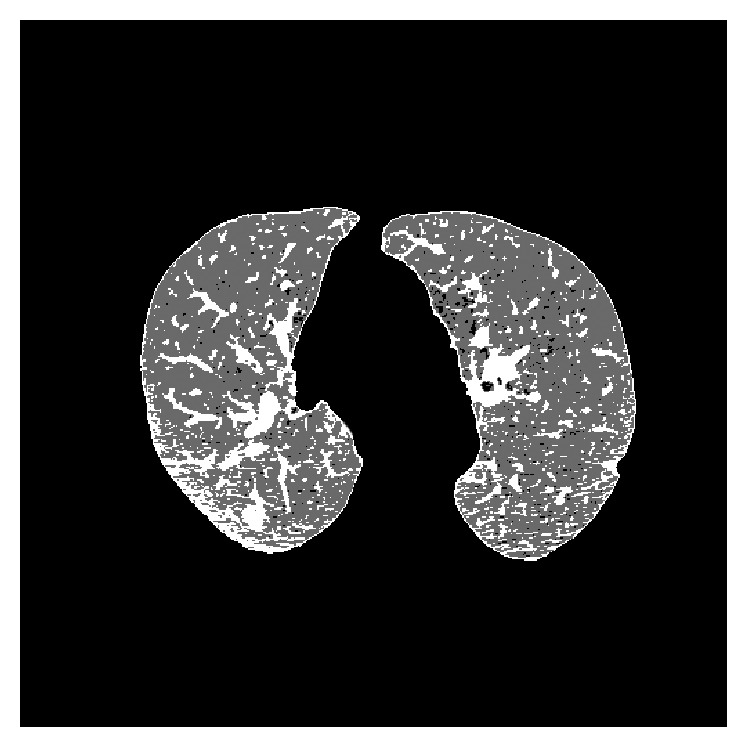
Image result for the KFCM algorithm.

The kernelized function employed by this algorithm is not able to discriminate between the pixels that belong to each cluster because of the overlap existing between the pixels in different clusters given that the only information that the algorithm uses is the attenuation value, which for the majority of pixels is very close for the nodule and the lung tissue. As such, this algorithm provides a good classification and a quality result for each slice or, if there are many serious errors, a low quality result. There is a substantial range in the success rate from 10 to 90%.  Figure 10 shows a slice result typical of the majority of cases. It can be seen that the algorithm correctly classifies almost all the pixels of the nodule but the number of false positives is very high complicating, to a large extent, the analysis in subsequent stages.

### 4.3. SFCM

**Figure 11:**
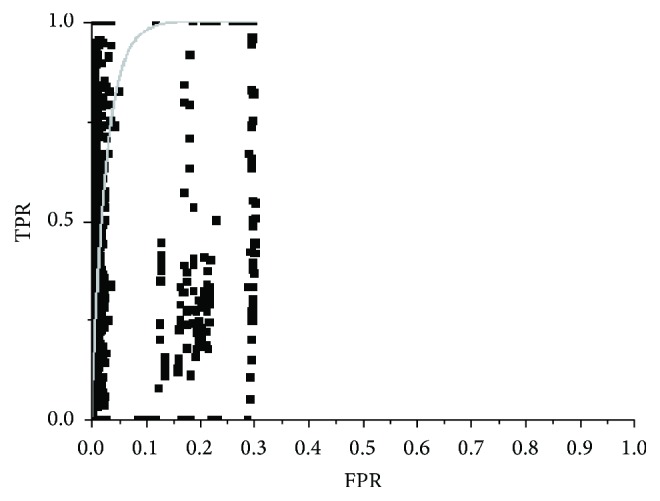
Results obtained for the SFCM algorithm.

**Figure 12:**
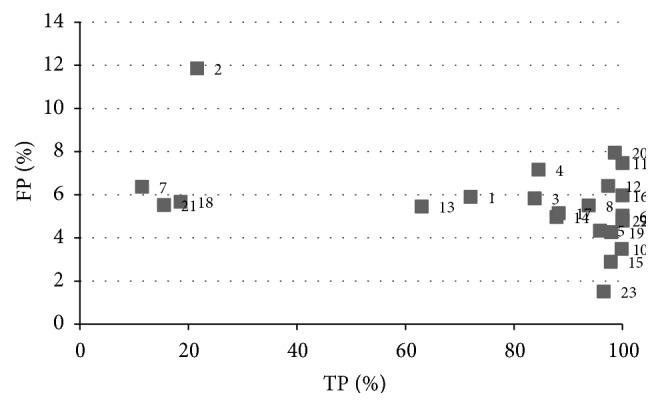
Results obtained for the SFCM algorithm.

The next algorithm to be analyzed was SFCM and Figure 11 shows the results of its application to the pool of test studies. What is notable about this algorithm, and clearly visible in the previous graph, is the low number of false positives produced (10–15% in almost slices). The reason for this result is the spatial character of the algorithm which makes it easier to differentiate (compared with the previously analyzed algorithms) the pixels which make up the nodule and those pixels which are part of the tissue when using the neighboring space to calculate membership. However this algorithm is unable of achieving a high success rate in the detection of the nodule in the majority of slices. It was only able to achieve an adequate level of success in about 30% of the slices which can be observed in Figure 12 in the distribution of points along the TP axis. This means that it is not a good option for the aim we have in mind in this paper, given that it cannot provide, with its high level of variability, a consistent rate of success for all the test studies.

Figure 12 shows the results obtained for the selected slices which are similar to those for the complete study: the number of false positives is low with a high success rate but clear variability depending on the slice. Selected samples were used in these tests. The slices with a low success rate were 2, 11, 18, and 7. The best results were obtained by partitioning the image in 3 clusters with the number of false positives less than if it was partitioned in 4 clusters without significantly reducing the true positives. However, the differences in the results were minimal when the only parameter varied was the samples: random or operator-selected.

**Figure 13:**
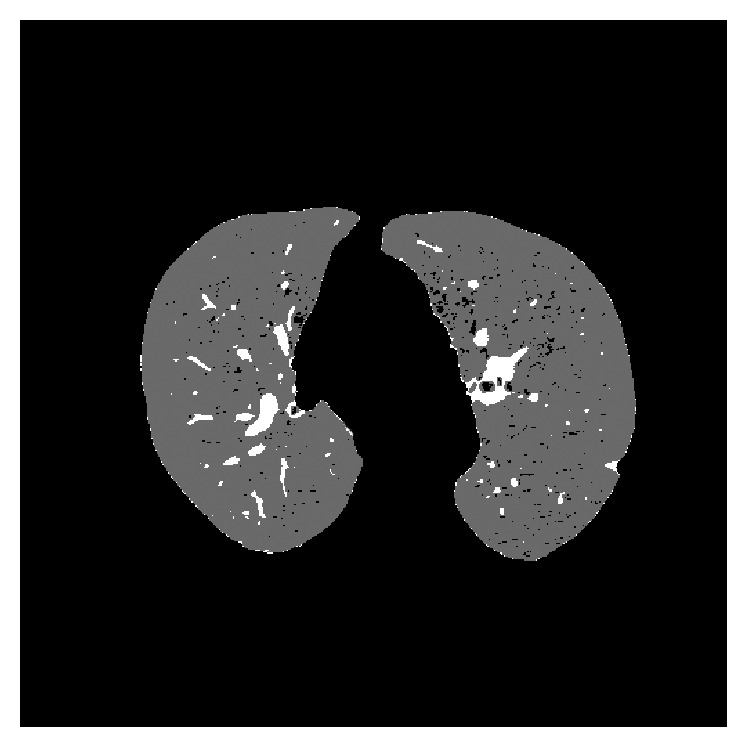
Resulting image for the SFCM algorithm.

This, in our view, does not indicate a limitation in this algorithm as it does in the FCM algorithm because, to obtain good results, it is necessary that the spatial function is the component with greater weight in the membership function. It is used as an additional characteristic to calculate the value of membership allowing the discrimination between pixels of different clusters based on neighborhood; so the more the importance it has, the less the number of false positives. This, however, causes the initial samples to have much less weight in the classification with the FCM membership much less valued and its influence on the final result much less.  Figure 13 shows a result for one of the test slices.

### 4.4. SKFCM

**Figure 14:**
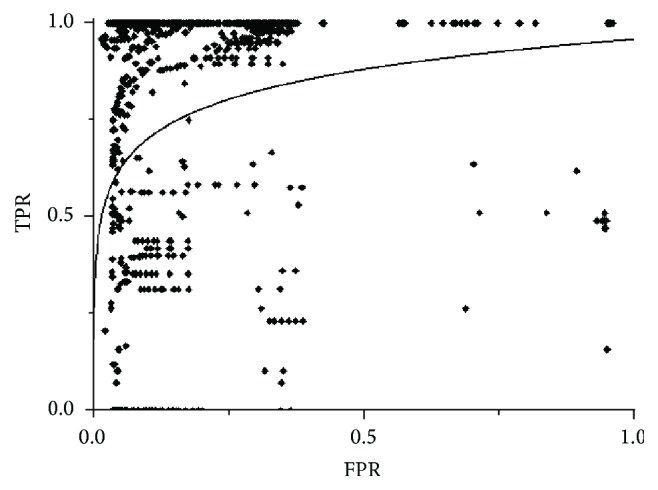
Results obtained for the SKFCM study set.

The results obtained for the algorithm SKFCM show a low level of false positives using selected samples. The best results were obtained by dividing the image into three clusters and using a spatial window 3 × 3; the success rate was above 80% for the majority of slices with the false positives lower than 20% for most of the study.  Figure 14 shows the results obtained for all the studies used in the analysis.

**Figure 15:**
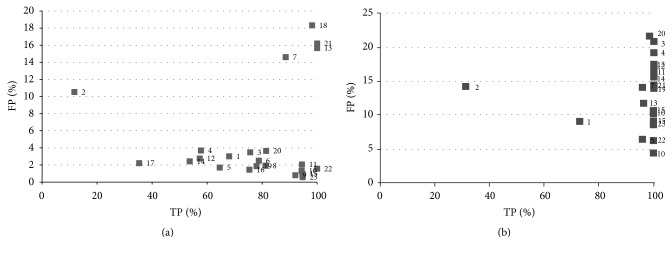
Results obtained for the SKFCM for the selected subset: (a) random samples, (b) operator samples.

The figure of true positives, using random samples, is grouped within the range of 60%–100%, although values of below 20% can be observed in some slices as, for example, slice numbered 2 (15% with random samples) (Figure 15). In the latter case, this results from the loss of pixels from the nodule during the preprocessing stage of the lungs and, above all, from the inability of the algorithm to divide the more complicated slices for classification.

**Figure 16:**
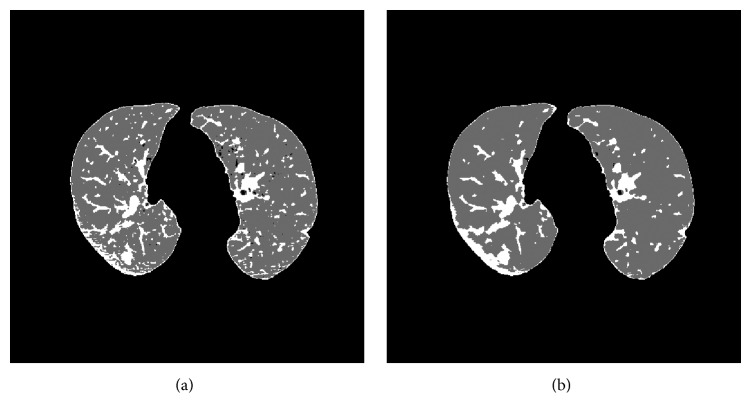
Results obtained for the algorithm SKFCM using different sigmas: (a) *σ* = 250, (b) *σ* = 500.

The most critical entry parameter for this algorithm is the sigma selection (Figure 16), obtaining significant variations in the results for the false positives varying this parameter, creating associated problems in the ROI classification at the next stage, and making identification of nodules difficult (Figure 16(b)).

In Figure 15, the results, using random and selected samples, can be observed having good ratios of true positives of around 100% for the greater part of the study using operator samples, although they do present a greater number of false positives with respect to using random samples. In the latter case, it can be seen that the success rate decreases for some images, to a range of between 50 and 100%.

The size of the neighboring window has not produced significant variations with its best value as indicated previously. This is due to the membership function having a strong dependence on the kernel function, which is strongly influenced by the initially selected samples.

### 4.5. MSKFCM

Finally, we will analyze the results of the new algorithm we are proposing which combines the two previous strategies, the objective of which is to improve classification using a kernelized function and to decrease the false positives taking into account the spatiality of each pixel. This is the trend that the most recent algorithms follow.

**Figure 17:**
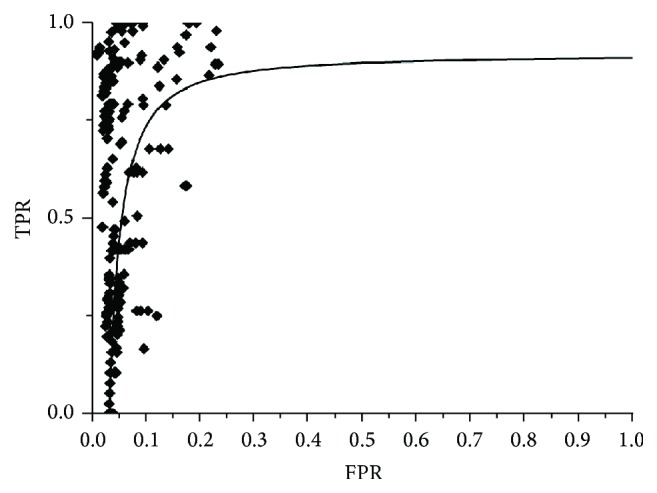
Results obtained in all studies for the MSKFCM algorithm.

**Figure 18:**
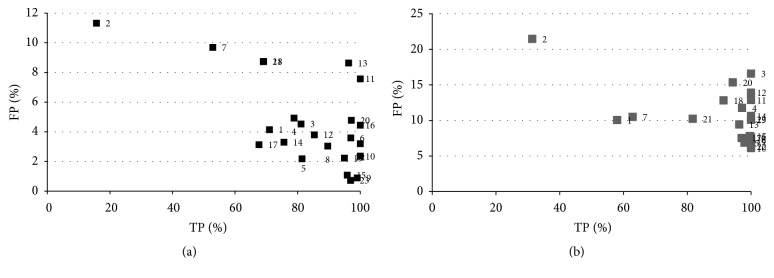
Results obtained for the MSKFCM for the selected subset: (a) random samples, (b) operator samples.

It can be observed in Figure 18 how the algorithm achieves a good success rate for almost all images with a low number of false positives (the worst result around 15%). This behaviour can also be seen in Figure 17 where the number of false positives has decreased substantially compared to other algorithms analyzed, maintaining a high success rate (>60%) for the majority of slices. It should be pointed out that although the curve is similar to that of SKFCM, this is due, not to the similar behaviour of the algorithm, but to the fitting function used.

**Figure 19:**
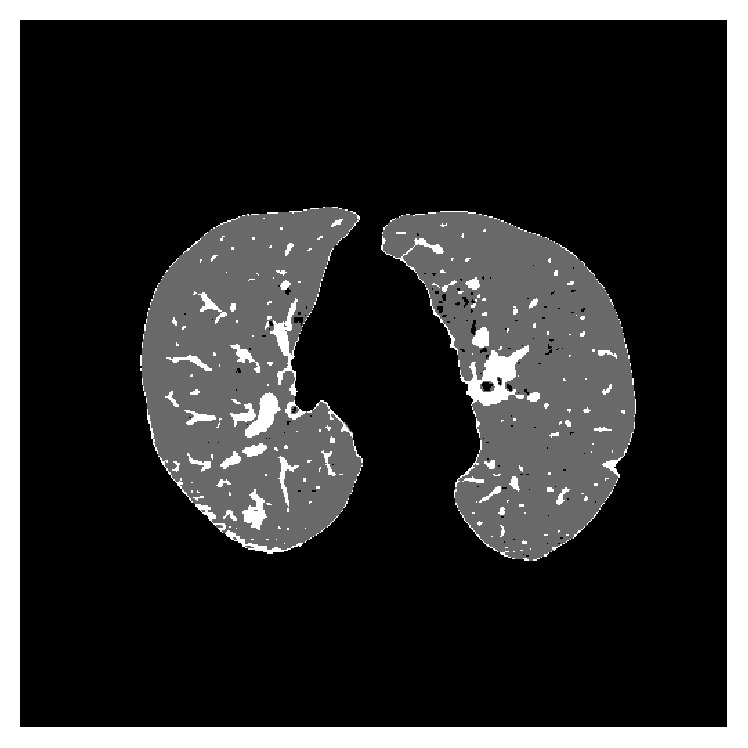
Results obtained for the algorithm MSKFCM for one of the slices using random samples.

By analyzing more in detail the results of the selected set of slices, the success rate deteriorated in the case of random samples (≈10%); we can also see a low rate of false positives was maintained, and in some cases improved results were obtained (Figure 17). Individually examining each slice with a low rate success, it can be seen that the lost part of the nodule in the majority of the slices could later be recovered using other techniques.  Figure 19 shows an example of a result applying this algorithm.

This algorithm also displays a more stable performance than the others (Table 2). A problem observed with the other algorithms is that when the sample set was modified in order to improve the results, there was also a variation in the cluster to which the nodule was assigned, depending on the initialisation and the number of clusters into which the slice had been divided. In the case of the new algorithm, however, when the number of clusters is set at 3, it consistently classifies the nodule in the same cluster, enabling, in addition to a good and stable performance with random samples, automated classification, which was the objective outlined at the beginning of this paper.

### 4.6. 3D Neighborhood

From the analysis of the results, it can be deduced that the algorithms which best address the problem presented in this paper are those which use spatial membership functions and, among these, those which combine this technique with a kernelized membership. To improve these results, we decided to modify the spatial kernelized algorithms to use 3D instead of 2D neighborhoods in the calculation of the memberships.

Helical thoracic CT scans allow for a 3D reconstruction of the target zone that is very similar to the original, given the high levels of resolution it is able to achieve. The use of the 3D structure instead of 2D provides more information when calculating memberships and avoids noise and loss of information associated with projecting a 3D structure in 2D.

**Figure 20:**
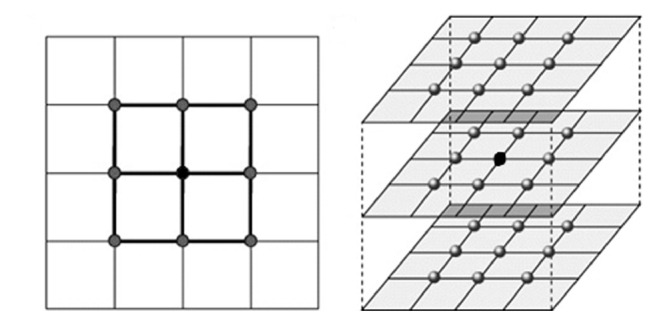
2D and 3D neighborhoods.

This modification was applied to those algorithms which provided the best results and presented more stable behaviour during the analysis: SKFCM and MKSFCM.  Figure 20 presents the scheme followed to obtain 3D neighborhood and the pixels that are used to calculate the spatial function for a 3 × 3 × 3 neighborhood, formed by the slice that the pixel belongs to; the previous and following in the form of a rectangular prism. It should be noted that, in its implementation using ITK, any shape (spherical, rhomboid) can be used to obtain the neighborhood.

The methodology, described for the test process in Section 3, was applied and in order to allow a direct comparison of the results, which were obtained in the same way from the same set of images, the test unit was the study and not the slice. For the two algorithms, tridimensional neighborhoods sized 3, 5, and 7 were used in rectangular prism. The parameters modified for the MSKFCM algorithm were *σ* in [150,700] and *p* and *q* in [1,2]. For the SKFCM the modified parameters were *σ* in [150,700] and *α* in [0.1,0.2]. The analysis of the results was conducted by slice for direct comparison with those obtained in previous tests.

The success rate, in the results obtained for the 3D version of MSKFCM, was close to 100% in more than 90% of the slices analyzed and the false positives did not exceed 18% in any of the slices. The slices that had a low success rate were juxtapleural nodules with problems, at the initial preprocessing stage, in maintaining all the points that belong to the nodule and nodules marked with a single pixel and classified as having an indefinite nature in the database. As such, and not being able to identify them as a nodule or not, they were of no interest to the present study.

It is worth noting in the results that using larger neighborhoods reduced the number of TPs and FPs until, in extreme cases, the algorithm does not detect any pixel as belonging to the nodule. The best results for success rates and greater stability were obtained using 3D neighborhoods sized 3 × 3 × 3.

The success rate for 3D SKFCM was similar to the previous algorithm at around 100%. However, the FP figure was high exceeding 60% in the poorest results. In addition, stability was low with a lot of variabilities in the results for different slices and the same slice with different parameters. The best results were obtained using small *α*, reducing the weight of the spatial factor. The behaviour of this algorithm is opposite to that of 3D MSKFCM: the greater the size of the neighborhood, the more the TPs and FPs increased.

For both algorithms, it was proven that the greater the size of the neighborhood, the greater the tendency of the algorithms to classify all the points in one cluster; the 3D distribution of the points does not correspond with the anticipated shape by the membership function (the membership function of the algorithms is based on FCM) resulting in an accumulation of errors in the classification.

The SKFCM algorithm tends to group all the pixels in the cluster identified as a nodule, because, with most important factor being the initial samples or centroids (in this case, the pixels which have been identified as belonging to the nodule are prioritized), the accumulation of errors means that more and more pixels associate with this cluster. MKFSCM, for its part, groups all the pixels in one cluster, identified as a lung, giving more weight to those pixels which form part of the neighborhood than to the centroids in the calculation of memberships, as the majority of the pixels in the slice are lung owing to the preprocessing which seeks to eliminate all elements of no interest; therefore, all of the pixels end up being assigned to this cluster.

Table 3 shows the results for the two algorithms for a combination of slices (for this table, different slices have been used from those used in Tables 2 and 4), selected from the nine studies using 2D and 3D neighborhoods; those that best reflect the behaviour of all the set have been chosen. The most notable aspect of all the results obtained is that the two types of neighborhoods are similar for the majority of cases. This is due to the spatial functions having been designed for work with 2D neighborhoods, unable to benefit from the additional information provided with the use of 3D neighborhoods.

As such, the algorithm which provides the best results using 3D neighborhoods is MKSFCM, the results of which are similar to the 2D algorithm with an improvement in results in only some slices.

## 5. Discussion

The most complicated pixels to classify correctly are those which belong to less well defined nodules, still at a very initial stage or juxtapleural, which are very difficult to distinguish from other pulmonary elements.

This was confirmed using the first version of the masks for the studies provided by the LIDC. Each pixel in these masks was assigned a value between 0 and 1000, representing the level of consensus among radiologists that the pixel under consideration belonged to a nodule (1000 indicates that all radiologists are in agreement with the membership of the pixel to a nodule and 0 that all were in agreement that it did not belong to a nodule). For those pixels, where there was strong agreement among the radiologists over membership to a nodule (with a punctuation equal to or above 800 points) both SKFCM and MSKFCM were capable of detecting them without any problems.

**Figure 21:**
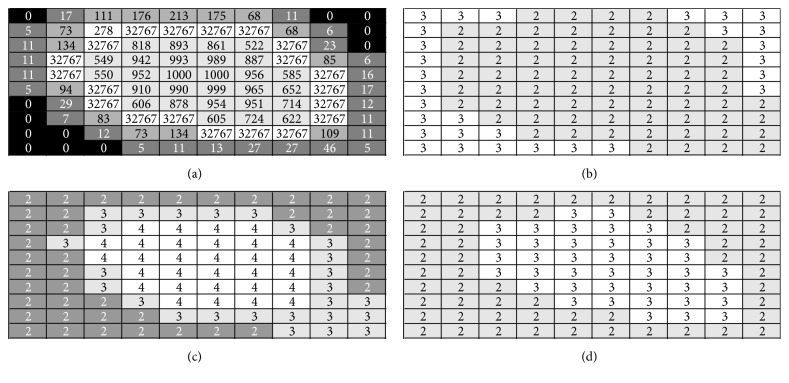
The best classification obtained for one of the images analyzed: mask (a), KFCM ((b), value 2 represents the nodule), SFCM ((c), value 4 represents the nodule), and MKSFCM ((d), value 3 represents the nodule).

Figure 21 shows a section of a slice classified as a nodule by radiologists and the different results provided by the algorithms which have been considered in this study. The majority of classification errors correspond to pixels with a low punctuation (100–200) especially those at the edge of the nodule.

The best results and performance of the MKSFCM algorithm were obtained by dividing the slices into three clusters. This was because the membership function with a larger number of classes is unable to divide the space of the problem in which the nodule pixels are clearly separated from the pixels belonging to other clusters; the cluster to which they are assigned depends on the distribution of coefficients in the image and the number of clusters into which the image is to be divided. A much more powerful membership function, capable of performing a better classification, would be required to obtain better results with a larger number of clusters.

This conclusion is corroborated by the fact that when using 3D neighborhoods which use more information and better reflect the structure of the element, the results do not present an improvement (Table 3) and maintain their level of success and, in some cases, increase the false positives. The best results were also obtained by dividing the image into three clusters.

## 6. Conclusions

This paper presents an extensive and thorough analysis of the use of traditional and state-of-the-art (Table 4) fuzzy clustering algorithms for detecting ROIs in helical thoracic CT slices, with the aim of incorporating this method into a CAD system that will help professionals to detect pulmonary nodules, tested using a set of studies selected from a public database.

Traditional algorithms have been shown not to be the most appropriate solution due to the limitations of the membership functions they use; they are unable to achieve good quality results with large sets of slices.

To resolve this limitation, algorithms which incorporate different modifications to the membership function were also analyzed: kernelized and spatial. The former improve the classification but continue to produce an elevated number of false positives, complicating the next stage of classification. Spatial algorithms also improve results but are very sensitive to noise (in the present case) and quite unstable given the significant variations, depending on the parameters and slice, in the results.

The next step was to analyze if by combining both techniques we could improve the results. We analyzed an algorithm which uses this strategy SKFCM and created a new algorithm combining two of the algorithms already analyzed: MSKFCM. Only by combining the two classification techniques (kernelized and spatial) was it possible to consistently classify the pixels as belonging to a nodule and therefore use it for the automatic detection of nodules in helical thoracic CT scans.

Both the SKFCM and MSKFCM algorithms provided an adequate rate of success for this task, with better results being obtained in some slices with the SKFCM algorithm. However, the MKSFCM algorithm presented more stable behaviour than the SKFCM algorithm with a much smaller number of false positives, allowing quality results to be obtained with a fixed set of input parameters and using samples not selected by an operator. This characteristic makes it more suitable for our objective: the automatic detection of ROIs that may be classified as a nodule.

## Figures and Tables

**Table 1:** Parameters analyzed and most relevant values identified for each parameter.

FCM	Number of clusters	[3,4]
	Set of initial samples	Random
		Operator

KFCM	Number of clusters	[3,4]
	Set of initial samples	Random
		Operator
	*σ*	[150,750]

SFCM	Number of clusters	[3,4]
	Set of initial samples	Random
		Operator
	*p*	[0,2]
	*q*	[0,2]

SKFCM	Number of clusters	[3,4]
	Set of initial samples	Random
		Operator
	*σ*	[150,750]

MSKFCM	Number of clusters	[3,4]
	Set of initial samples	Random
		Operator
	*p*	[0,2]
	*q*	[0,2]
	*σ*	[150,750]

**Table 2:** Results (%) for a subset of slices displaying the greatest problems for the algorithms using spatial information.

		SFCM			SKFCM			MSKFCM		
		3 × 3	5 × 5	7 × 7	3 × 3	5 × 5	7 × 7	3 × 3	5 × 5	7 × 7
1	TP	72.0	72.0	71.0	73.0	73.0	66.0	64.0	58.0	56.0
	FP	5.9	5.5	5.1	9.1	9.7	12.6	9.5	9.7	9.4

2	TP	21.6	19.6	19.6	31.4	33.3	33.3	29.4	29.4	29.4
	FP	11.9	11.7	11.6	14.3	17.3	18.5	21.2	21.3	21.1

7	TP	11.4	8.6	5.7	95.7	95.7	94.3	61.4	64.3	60.0
	FP	6.4	5.9	5.4	14.0	12.4	10.3	9.9	10.2	9.7

13	TP	62.9	59.3	55.6	96.3	100.0	96.3	96.3	100.0	92.6
	FP	5.5	4.9	4.6	11.8	11.2	10.2	8.9	10.1	8.9

17	TP	88.2	88.2	85.3	100.0	100.0	100.0	97.1	97.1	97.1
	FP	5.1	4.8	4.6	10.4	10.7	9.8	6.6	6.8	7.0

21	TP	15.5	8.4	5.6	100.0	100.0	100.0	69.0	81.7	81.7
	FP	5.5	4.9	4.5	14.4	11.3	9.0	8.5	9.9	9.4

**Table 3:** Results for slices from different studies using 2D and 3D neighborhoods.

			3 × 3		5 × 5		7 × 7	
			2D	3D	2D	3D	2D	3D
1	MKSFCM	VP	61.8	61.8	43.6	60	41.8	50.9
		FP	6.9	7.3	3.8	6.3	3.6	5.9
	SKFCM	VP	49.1	63.6	49.1	63.6	60	63.6
		FP	5	34.2	5.2	29.4	5.8	31.3

2	MKSFCM	VP	30.9	49.4	27.2	45.7	23.5	42
		FP	3.3	5.7	3.1	5	2.9	4.4
	SKFCM	VP	34.6	51.9	34.6	51.9	35.8	53.1
		FP	3.7	26.3	3.7	29.6	3.70	31.1

3	MKSFCM	VP	100	95.7	65.2	30.4	47.8	21.7
		FP	4.8	3.6	3.8	1.9	1.8	1.7
	SKFCM	VP	95.7	100	100	100	100	100
		FP	3.5	10.1	8	19.2	14.9	23.4

4	MKSFCM	VP	100	95	95	95	90	95
		FP	9.4	6.3	6	5.5	5	5.3
	SKFCM	VP	100	100	100	100	100	100
		FP	30.8	23.8	32.8	26.6	34.3	41.7

5	MKSFCM	VP	78.9	78.9	78.9	78.9	0	76.3
		FP	13.6	13.7	9.5	13.9	3	14.5
	SKFCM	VP	89.5	89.5	89.5	89.5	89.5	92.1
		FP	20.9	35.2	26.1	38.5	28.7	40.1

6	MKSFCM	VP	96.9	90.70	93.8	88.7	85.6	67
		FP	17.4	6.7	15.8	4.3	15.7	3.7
	SKFCM	VP	97.9	97.3	99	97.9	99	97.9
		FP	31.1	21.3	33.5	27.5	35.3	30.2

7	MKSFCM	VP	43.8	43.8	42.2	42.2	42.2	32.8
		FP	7	6.8	6.2	5.5	5.3	4.7
	SKFCM	VP	43.8	43.8	43.8	43.8	43.8	43.8
		FP	9.7	8.4	15.3	15.9	21	21.2

8	MKSFCM	VP	98.8	97.6	97.6	84.5	100	84.5
		FP	4.6	3.8	3.6	2.6	4.4	2.4
	SKFCM	VP	100	100	100	100	100	100
		FP	6	5.2	8.6	10.1	11.7	14.2

9	MKSFCM	VP	97.8	95.1	92.2	92.1	91.6	91.6
		FP	5.5	1.7	1.1	1.1	0.9	0.9
	SKFCM	VP	97	97.8	100	100	100	100
		FP	2.4	5.9	13	14.7	17.1	19.2

**Table 4:** Results (%) for a subset of slices which present greater problems for the different algorithms.

		FCM	SFCM	KFCM	SKFCM	MKSFCM
1	TP	72	72	73	73	64
	FP	6.4	5.9	15.4	9.7	9.5

2	TP	23.5	21.6	35.3	33.3	29.4
	FP	12.2	11.9	25.7	17.3	21.2

7	TP	27.1	11.4	98.6	95.7	61.4
	FP	7.9	6.4	19.5	12.4	9.9

13	TP	77.8	62.9	100	100	96.3
	FP	7.1	5.5	18.4	11.2	8.9

17	TP	94.1	88.2	100	100	97.1
	FP	5.9	5.1	13.2	10.7	6.6

21	TP	38	15.5	100	100	69
	FP	7.5	5.5	23.9	11.3	8.5
